# Dissemination of Multidrug-Resistant Commensal *Escherichia coli* in Feedlot Lambs in Southeastern Brazil

**DOI:** 10.3389/fmicb.2019.01394

**Published:** 2019-06-25

**Authors:** Katia Suemi Gozi, Juliana Rodrigues Froes, Luana Perpetua Tobias Deus Ajude, Caroline Rodrigues da Silva, Rafaela Speranza Baptista, Juliana Regina Peiró, Marcia Marinho, Luiz Claudio Nogueira Mendes, Mara Corrêa Lelles Nogueira, Tiago Casella

**Affiliations:** ^1^Centro de Investigação e Microrganismos, FAMERP, São José do Rio Preto, Brazil; ^2^Faculdade de Medicina Veterinária, São Paulo State University (UNESP), Araçatuba, Brazil; ^3^Hospital de Base, São José do Rio Preto, Brazil

**Keywords:** *Escherichia coli*, sheep, multidrug resistance, cephalosporin, aminoglycoside, tetracycline, trimethoprim/sulfamethoxazole, phenicols

## Abstract

Antimicrobial resistance (AR) is a public health issue since it limits the choices to treat infections by *Escherichia coli* in humans and animals. In Brazil, the ovine meat market has grown in recent years, but studies about AR in sheep are still scarce. Thus, this study aims to investigate the presence of AR in *E. coli* isolated from lambs during feedlot. To this end, feces from 112 lambs with 2 months of age, after weaning, were collected on the first day of the animals in the feedlot (day 0), and on the last day before slaughtering (day 42). Isolates were selected in MacConkey agar supplemented with 4 mg/L of ceftiofur and identified by biochemical methods. Isolates were submitted to an antimicrobial susceptibility test by disc-diffusion and PCR to investigate genes for phylogenetic group, virulence determinants and resistance to the several antimicrobial classes tested. The genetic localization of the *bla* genes detected was elucidated by S1-PFGE followed by Southern blot-hybridizations. The isolates were typed by *XbaI*-PFGE and MLST methods. Seventy-eight *E. coli* were isolated from 8/112 (7.1%) animals on day 0, and from 55/112 (49.1%) animals on day 42. Since only *fimH* was present in almost all *E. coli* (97.4%) as a virulence gene, and also 88.5% belonged to phylogroups B1 or A, we consider that isolates represent intestinal commensal bacteria. The dendrogram separated the 78 non-virulent isolates in seven clusters, two of which comprised 50 *E. coli* belonging to ST/CC 1727/446 or ST 3994 recovered on day 42 commonly harboring the genotype *bla*_CMY -2_-*aac(3)-IIa* -*tetA*-*sul1*-*sul2*-*floR*-*cmlA*. Special attention should be given to the presence of *bla*_CTX-M-15_, a worldwide gene spread, and *bla*_CTX-M-14_, a hitherto undetected gene in *Enterobacteriaceae* from food-producing animals in Brazil. Importantly, *E. coli* lineages and plasmids carrying *bla* genes detected here have already been reported as sources of infection in humans either from animals, food, or the environment, which raises public health concerns. Hence, two types of commensal *E. coli* carrying important AR genes clearly prevailed during feedlot, but lambs are also reservoirs of bacteria carrying important AR genes such as *bla*_CTX-M-14_ and *bla*_CTX-M-15_, mostly related to antimicrobial treatment failure.

## Introduction

The use of antimicrobial agents in humans and animals can cause the emergence and dissemination of antimicrobial resistance (AR) in pathogens, which may compromise the effective treatment of infections in humans (Kaesbohrer et al., 2012). International public health agencies have reported the potential link and risks between the overuse or misuse of antimicrobials in veterinary practices and the emergence of human resistant pathogens, which encourage surveillance of AR and antimicrobial use worldwide (EFSA, 2011; WHO, 2017). Human exposure to AR bacteria through direct contact with animals, consumption and handling of contaminated food, and bacteria released into the environment may contribute to the spread of AR determinants (Kaesbohrer et al., 2012).

Infections caused by AR *E. coli* and their isolation from food-producing animals are increasing worldwide (EFSA, 2008, 2011; Kaesbohrer et al., 2012). This scenario is regarded as a consequence of the selective pressure exerted on the gastrointestinal tract (GIT) of the animals by the overuse of antimicrobials (Graham et al., 2017). During slaughtering, the carcass may be contaminated and AR commensal or pathogenic bacteria might reach humans through the food chain (Cyoia et al., 2019; Projahn et al., 2019). The relationship between AR strains isolated from humans and the food chain has been already reported (Belmar Campos et al., 2014). Therefore, the monitoring of commensal bacteria is important since it constitutes a reservoir of AR genes, which allows the tracking of emerging resistance in livestock and possible spread to animal-derived food and other zoonotic pathogens (EFSA, 2008; Kaesbohrer et al., 2012; Madec and Haenni, 2018).

The majority of studies about antimicrobial use and resistance in food-producing animals are carried out on cattle, chickens, and pigs, but in regard to other food-producing flocks, such as sheep, information is scarce. Little is known about AR in sheep in Brazil, despite the increased consumption of lamb meat (FAO, 2018). Therefore, this study aims to determine the distribution of AR *E. coli* in the fecal microbiota of feedlot lambs in Brazil.

## Materials and Methods

### Study Population

A special feedlot comprising 140 lambs with 2 months of age, after weaning, coming from 35 different farms in the State of São Paulo, Southeastern Brazil was chosen for this study. Stool samples were collected weekly from the rectum of the animals for parasitological screening between September 14, 2016 and October 27, 2016, under the Ethics Committee approval number FOA00845-2017. Trimethoprim/sulfamethoxazole was used to prevent and to treat clinical manifestations of respiratory disease, and florfenicol was used to treat infectious keratoconjunctivitis. Stool samples from 112 lambs were collected immediately after the arrival of the sheep at the feedlot (day 0) and then on the day before the slaughtering of the animals (day 42) to further investigate the presence of AR *E. coli*.

### Bacterial Culture, Identification and Antimicrobial Susceptibility

About one gram of feces was diluted in 5 mL of sterile NaCl 0.9% and directly inoculated onto MacConkey agar (Oxoid) supplemented with 4 mg/L of ceftiofur (Lapisa). Following incubation at 37°C for 18–24 h, one of each of the different presumptive *E. coli* colonies (i.e., pinkish round colony due to lactose fermenting, dry to little mucous aspect, and characteristic odor) were selected for identification by biochemical essays using a commercial kit (NewProv) and further characterization described below.

Antimicrobial susceptibility testing was performed following the Clinical and Laboratory Standards Institute (CLSI, 2017) guidelines using the disc diffusion method. Bacterial susceptibility to 13 beta-lactam and non-beta-lactam antibiotics (Oxoid) of veterinary and human interest was tested: amoxicillin/clavulanate, ceftazidime, cefotaxime, ceftiofur, cefoxitin, ertapenem, amikacin, gentamicin, enrofloxacin, nalidixic acid, tetracycline, trimethoprim/sulfamethoxazole, florfenicol, and chloramphenicol. Parallel to the antimicrobial susceptibility test, the phenotypic test for production of extended-spectrum beta-lactamase was performed by the Modified Double Disc Synergy Test (Kaur et al., 2013). *E. coli* ATCC 25922 and *Klebsiella pneumoniae* ATCC 700603 were used as quality control strains.

### AR and Virulence Genes, and Phylogenetic Grouping

Investigation of the main plasmidial genes associated with cephalosporins resistance (*bla*_CTX-M_ and *bla*_CMY_), aminoglycosides resistance [*aac(3)-Ia*, *aac(3)-IIa*, *acc(6*′*)-Ih*, *ant(2*″*)-Ia*, *aph(3*′*)-VI*, *aph(3*′*)-Ia* and *aac(6*′*)-Ib*], quinolones resistance (*qnrA*, *qnrB*, *qnrC*, *qnrD*, *qnrS*, *qepAB*, and *oqxAB*), tetracycline resistance (*tetA*, *tetB*, and *tetC*), trimethoprim resistance (*dfr Ia*, *dfr VII*, and *dfr XII*), sulphas resistance (*sul1* and *sul2*), and phenicols resistance (*floR*, *cat* and *cmlA*) was performed in each respective resistant strain by PCR according to previous protocols (Supplementary Table 1). Products of *bla* genes were sequenced using the corresponding primers in order to identify the variant detected and analyzed using BLAST^1^.

The following 20 virulence genes, which have been associated with Extraintestinal Pathogenic *E. coli* strains, were investigated by PCR as previously described (Supplementary Table 1): *fimH*, *papEF*, *papG I*, *papG II*, *papG III*, *sfa/focDE*, *sfaS*, *focG*, *afa/draBC*, *nfaE*, *kpsMT K1*, *kpsMT K5*, *hlyA*, *cnf1*, *cdtB*, *sat*, *vat*, *fyuA*, *iutA*, and *iroN*. Since sheep are known as an important source of Shiga-toxin producing-*E. coli* (STEC) (Vettorato et al., 2009), the *stx1* and *stx2* genes, as well as the *aggR* and the *eae* genes, associated with Enteroaggregative *E. coli* (EAEC) and Enteropathogenic *E. coli* (EPEC), respectively, were additionally searched by PCR according to previous protocols (Supplementary Table 1). *E. coli* isolates were also submitted to phylogenetic grouping for predicting of commensal or pathogenic isolates as previously described (Clermont et al., 2000; Doumith et al., 2012).

### Plasmids Typing

Replicon of the plasmids of the isolates was detected by PCR-based Replicon Typing scheme (Carattoli et al., 2005; Villa et al., 2010) using the PBRT 2.0 kit (DIATHEVA). S1 enzyme (Promega) was used for 45 min to linearize plasmids and results were visualized in *Pulsed-Field Gel Electrophoresis* (S1-PFGE) for 20 h with initial switch time = 1 s and final switch time = 30 s on an electric field of 6 V/cm. Southern blot-hybridization analysis on S1-PFGE gels was performed using adequate probes and the kits Amersham^TM^ AlkPhos Direct Labeling Reagents and Amersham^TM^ CDP-Star^TM^ Detection Reagent (GE Healthcare).

### Isolates Typing

Bacterial DNA was typed by restriction with *Xba*I (Thermo Scientific) followed by a PFGE (*Xba*I-PFGE) for 22 h with initial switch time = 2.2 s and final switch time = 54.2 s, and 6 V/cm. The software BioNumerics^TM^ version 7.6.3 (Applied Maths) was used for dendrogram construction and clustering based on the band-based Dice’s similarity coefficient and the unweighted pair group method using arithmetic averages. Isolates were considered to belong to the same cluster when the similarity coefficient was ≥90%.

*Escherichia coli* isolates were additionally submitted to *Multilocus Sequence Typing* according to the Achtman’s scheme^2^.

### Nucleotide Sequence Accession Number

The *bla* genes sequences reported in this study have been deposited to GenBank under accession numbers MK896925 to MK896944 and MK917695 to MK917713.

## Results

**FIGURE 1:**
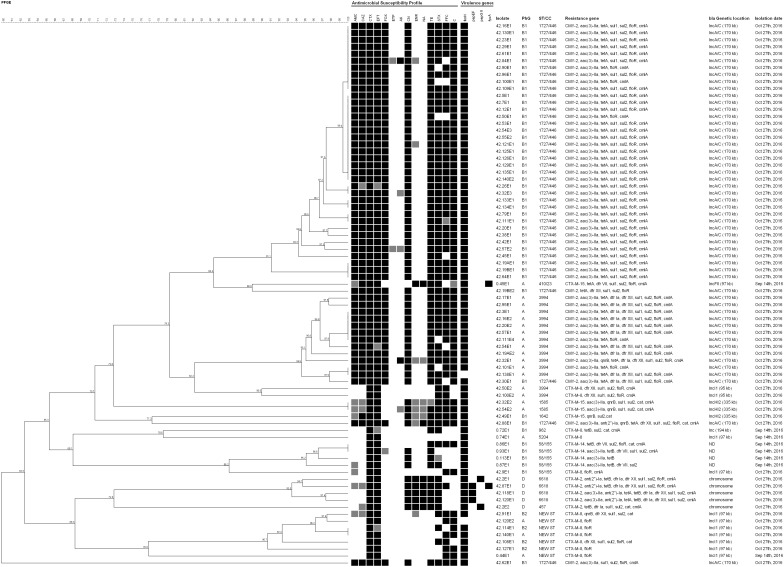
Dendrogram obtained from *Xba*I-PFGE typing of the 78 *E. coli* isolated. Dendrogram was constructed using Optimization 0% and Tolerance 1.5%. AMC, amoxicillin/clavulanate; CAZ, ceftazidime; CTX, cefotaxime; EFT, ceftiofur; FOX, cefoxitin; ETP, ertapenem; AK, amikacin; CN, gentamicin; ENR, enrofloxacin; NA, nalidixic acid; TE, tetracycline; STX, trimethoprim/sulfamethoxazole; FFC, florfenicol; C, chloramphenicol. Antimicrobial Susceptibility Profile squares: black, resistance; gray, intermediate resistance; white, susceptibility. Virulence genes squares: black, presence; white, absence. PhG, phylogenetic group. ST/CC, Sequence Type/Clonal Complex. ND, localization not detected. Isolation dates “Sep 14th, 2016” and “Oct 27th, 2016” refers to “day 0” and “day 42,” respectively.

Eight CTX-M-producing *E. coli* were isolated from eight animals on day 0, and 70 CTX-M- or CMY-2-producing *E. coli* were isolated from 55 lambs on day 42 (Figure 1 and Table 1). All 78 isolates presented resistance to at least one of the third-generation cephalosporins – 3GC tested (ceftazidime, cefotaxime, ceftiofur). The 53 CMY-2-producing *E. coli* presenting resistance to amoxicillin/clavulanic acid also presented resistance to the cephamycin cefoxitin (Figure 1). More than 80% of the isolates presented additional resistance to at least one of the phenicols tested (68, 87.2%), to tetracycline (66 isolates, 84.6%), to trimethoprim/sulfamethoxazole (65, 83.3%), and at least one of the aminoglycosides tested (64, 82.0%). Only seven isolates (9.0%) presented resistance to nalidixic acid and/or enrofloxacin, and all *E. coli* were susceptible to ertapenem (Table 1).

**Table 1:** Isolates presenting resistance to each antimicrobial class among the 78 *E. coli* from stools of sheep in Southeastern Brazil.

Antimicrobial class	N isolates (%)	*bla* gene associated
Penicillin + beta-lactamase inhibitors	53 (68.0)	CMY-2
Third-generation cephalosporins	78 (100.0)	CTX-M-2, –8, –14, –15, CMY-2
Cephamycin	53 (68.0)	CMY-2
Aminoglycosides	64 (82.0)	CTX-M-2, –14, –15, CMY-2
Quinolones	7 (9.0)	CTX-M-2, –15, CMY-2
Tetracycline	66 (84.6)	CTX-M-2, –8, –14, –15, CMY-2
Folate pathway inhibitors	65 (83.3)	CTX-M-2, –8, –14, –15, CMY-2
Phenicols	68 (87.2)	CTX-M-2, –8, –14, –15, CMY-2


*The bla genes associated with each antimicrobial class resistance are also presented.*

In total, 18 genes responsible for antimicrobial resistance were detected in this study, and all of the 78 isolates presented *bla*_CMY -2_ or *bla*_CTX-M_ genes (Figure 1 and Table 2). The genes *bla*_CTX-M-8_, *bla*_CTX-M-14_, and *bla*_CTX-M-15_ were identified in the isolates from day 0 harbored by plasmids IncI1 of ∼97 kb or IncHI1 ∼194 kb for *bla*_CTX-M-8_, and plasmid FII of about 97 kb for *bla*_CTX-M-15_. We could not detect plasmids harboring *bla*_CTX-M-14_. The *bla*_CTX-M-2_, *bla*_CTX-M-8_, *bla*_CTX-M-15_, and *bla*_CMY -2_ were identified in isolates recovered on day 42 inserted into the chromosome in the case of *bla*_CTX-M-2_, and harbored by plasmids IncI1 of about 95 kb or 97 kb for *bla*_CTX-M-8_, plasmid IncHI2 of ∼335 kb for *bla*_CTX-M-15_, and plasmid IncA/C of ∼170 kb for all *bla*_CMY -2_ (Table 3). Regarding resistance to aminoglycosides, especially gentamicin, the *aac(3)-IIa* gene was detected in 60 isolates (76.9%) on days 0 and 42 of feedlot while the *ant(2*″*)-Ia* gene was detected only in five isolates (6.4%) on day 42. The *qnrB* gene was the only one detected as responsible for quinolone non-susceptibility, present in six isolates (7.7%) obtained on day 42. The *tetA* and *tetB* genes, responsible for tetracycline resistance, were detected in 54 (69.2%) and 10 (12.8%) isolates, respectively, on days 0 and 42 of feedlot. Concerning resistance to trimethoprim, the *dfr VII* gene was detected only on day 0 of feedlot in four isolates (5.1%), and *dfr Ia* and *dfr XII* were detected only on day 42 in 16 (20.5%) and 21 (26.9%) isolates, respectively. Resistance to sulphas was detected at both the first and last days of feedlot, with 61 isolates (78.2%) carrying *sul1*, and 65 (83.3%) carrying the *sul2* gene. Lastly, in regard to phenicols resistance, the *floR* and the *cmlA* genes were detected in 65 (83.3%) and 67 (85.9%) isolates, respectively, while the *cat* gene was detected in only eight isolates (10.3%); all recovered on both days 0 and 42 of feedlot (Figure 1 and Table 2).

**Table 2:** Antimicrobial resistance genes distribution among the 78 *E. coli* from stools of sheep in Southeastern Brazil, according to the animals (ID) and the day of feedlot they were detected.

Antimicrobial class	Resistance gene	Distribution (%)	Animal ID (n)	Day
Third-	*bla*_CTX-M-2_	5 (6.4)	2, 87, 118, 120 (4)	42
generation	*bla*_CTX-M-8_	12 (15.4)	9, 44, 50, 72, 74, 91, 100, 108, 114, 120, 127, 140 (12)	0, 42
cephalosporins	*bla*_CTX-M-14_	4 (5.1)	86, 87, 93, 113 (4)	0
	*bla*_CTX-M-15_	4 (5.1)	32, 45, 49, 54 (4)	0, 42
	*bla*_CMY -2_	53 (68.0)	3, 5, 7, 12, 16, 17, 19A, 19B, 20, 23, 26, 29, 30, 32, 38, 42, 45, 50, 53, 54, 55, 57, 61, 62, 64, 79, 84, 88, 90, 95, 96, 100, 101, 109, 111, 121, 125, 126, 129, 130, 133, 134, 135, 138, 140 (45)	42
Aminoglycoside	*aac(3)-IIa*	60 (76.9)	2, 3, 5, 7, 12, 16, 17, 19A, 19B, 20, 23, 26, 29, 30, 32, 38, 42, 45, 49, 50, 53, 54, 55, 57, 61, 62, 64, 79, 84, 87, 88, 90, 93, 95, 96, 100, 101, 109, 111, 113, 118, 120, 121, 125, 126, 129, 130, 133, 134, 135, 138, 140 (52)	0, 42
	*ant(2*″*)-Ia*	5 (6.4)	2, 87, 88, 118, 120 (5)	42
Quinolone	*qnrB*	6 (7,7)	32, 49, 54, 88, 91 (5)	42
Tetracycline	*tetA*	54 (69.2)	3, 5, 7, 12, 16, 17, 19A, 19B, 20, 23, 26, 29, 30, 32, 38, 42, 45, 50, 53, 54, 55, 57, 61, 64, 79, 84, 88, 90, 95, 96, 100, 101, 109, 111, 120, 121, 125, 126, 129, 130, 133, 134, 135, 138, 140 (45)	0, 42
	*tetB*	10 (12.8)	2, 72, 86, 87, 93, 113, 118, 120 (8)	0, 42
Trimethoprim	*dfr Ia*	16 (20.5)	2, 3, 16, 17, 19A, 20, 30, 32, 54, 57, 87, 95, 118, 120, 138 (15)	42
	*dfr VII*	4 (5.1)	45, 86, 87, 93 (4)	0
	*dfr XII*	21 (26.9)	2, 3, 16, 17, 19A, 19B, 20, 30, 32, 50, 54, 57, 87, 88, 91, 95, 100, 108, 118, 120, 138 (21)	42
Sulphas	*sul1*	61 (78.2)	2, 3, 5, 7, 12, 16, 17, 19A, 19B, 20, 23, 26, 29, 30, 32, 38, 42, 45, 50, 53, 54, 55, 57, 61, 62, 64, 79, 84, 87, 88, 91, 93, 95, 96, 100, 108, 109, 111, 118, 120, 121, 125, 126, 129, 130, 133, 134, 135, 138, 140 (50)	0, 42
	*sul2*	65 (83.3)	2, 3, 5, 7, 12, 16, 17, 19A, 19B, 20, 23, 26, 29, 30, 32, 38, 42, 45, 49, 50, 53, 54, 55, 57, 61, 62, 64, 72, 79, 84, 86, 87, 88, 91, 93, 95, 96, 100, 108, 109, 111, 118, 120, 121, 125, 126, 129, 130, 133, 134, 135, 138, 140 (53)	0, 42
Phenicols	*floR*	65 (83.3)	3, 5, 7, 9, 12, 16, 17, 19A, 19B, 20, 23, 26, 29, 30, 32, 38, 42, 44, 45, 50, 53, 54, 55, 57, 61, 62, 64, 79, 84, 86, 87, 88, 90, 95, 96, 100, 101, 108, 109, 111, 114, 120, 121, 125, 126, 127, 129, 130, 133, 134, 135, 138, 140 (53)	0, 42
	*cat*	8 (10.3)	2, 32, 49, 54, 86, 88, 91, 108 (8)	0, 42
	*cmlA*	67 (85.9)	2, 3, 5, 7, 9, 12, 16, 17, 19A, 19B, 20, 23, 26, 29, 30, 32, 38, 42, 44, 45, 50, 53, 54, 55, 57, 61, 62, 64, 72, 79, 84, 86, 87, 88, 90, 93, 95, 96, 100, 101, 109, 111, 118, 120, 121, 125, 126, 129, 130, 133, 134, 135, 138, 140 (54)	0, 42


**Table 3:** Genetic localization of *bla* genes detected in the 78 *E. coli* isolates.

*bla* gene	Localization	N isolates	Day
CTX-M-2	chromosome	5	42
CTX-M-8	IncI1 (95 kb)	2	42
	IncI1 (97 kb)	9	0, 42
	IncHI1 (194 kb)	1	0
CTX-M-14	ND^∗^	4	0
CTX-M-15	IncFII (97 kb)	1	0
	IncHI2 (335 kb)	3	42
CMY-2	IncA/C (170 kb)	53	42


*The size of plasmids is described between parentheses and represents an approximation according to S1-PFGE gels and the molecular reference. ^∗^Not detected.*

Four virulence genes were detected, but only *fimH* was present in the majority (76 isolates, 97.4%). The *papEF* was detected in 3 isolates (3.8%), and *papG* II and *fyuA* in 2 (2.6%), as presented in Figure 1. Furthermore, five genotypes concerning virulence were detected, including the absence of any gene, the presence of only *fimH* or a combination of it and the other genes detected (Table 4). No genes predictive of STEC, EAEC or EPEC were detected. Forty-eight (61.5%) *E. coli* belonged to phylogroup B1, 21 (27.0%) to phylogroup A, 5 (6.4%) to phylogroup D, and 4 (5.1%) to phylogroup B2. Phylogroup D was related only to the *bla*_CTX-M-2_ gene (Table 4).

**Table 4:** Genotypes detected concerning virulence genes in the 78 *E. coli* isolates from stools of sheep in Southeastern Brazil.

	Virulence genotypes		N isolates	Phylogroup	*bla* gene	Animal	Day
–			2	A	CTX-M-8, CMY-2	#120, #138	42
*fimH*			70	A, B1, B2	CTX-M-8, –14, –15, CMY-2	all others	0, 42
*fimH*	*papEF*		2	D	CTX-M-2	#118, #120	42
*fimH*	*papG II*		2	D	CTX-M-2	#2	42
*fimH*	*fyuA*		1	A	CTX-M-15	#45	0
*fimH*	*papEF*	*fyuA*	1	D	CTX-M-2	#87	42


*Genotypes are described according to the number of isolates, the phylogenetic groups and the *bla* genes related to each one, and also according to ID of the animals (#) and the day in which the *E. coli* with each combination of virulence genes were isolated.*

The *Xba*I-PFGE typing distinguished the 78 isolates in seven major clusters. Two lambs (animals #45 and #87) presented AR *E. coli* (isolates 0.45E1 and 42.45E1, and 0.87E1 and 42.87E1, respectively) on both days 0 and 42, but the strains are not similar by *Xba*I-PFGE and neither carry the same AR genes (Figure 1). Typing with the MLST scheme revealed 10 Sequence Types (ST) of *E. coli* in the studied feedlot, and one new allele profile in seven *bla*_CTX-M-8_-carrying isolates (0.44E1, 42.91E1, 42.108E1, 42.114E1, 42.120E2, 42.127E1, and 42.140E1) recovered on the first and last days of feedlot. The ST 1727 Clonal Complex (CC) 446 was predominant and present only on day 42 in 52.6% of the total isolates regarding just *bla*_CMY -2_-carrying *E. coli* from phylogenetic group B1, followed by the ST 3994, the new combination of MLST alleles, ST/CC 58/155, ST 6618, ST 1585, and the ST/CC 410/23, ST 457, ST 962, ST 1642, ST 5204 (Table 5).

**Table 5:** Sequence Types and Clonal Complexes detected for the *E. coli* isolated from sheep in Southeastern Brazil.

ST/CC^a^	N isolates (%)	*bla* gene associated	Phylogroup	Day
58/155	5 (6.4)	CTX-M-8, CTX-M-14	B1	0
410/23	1 (1.3)	CTX-M-15	A	0
457	1 (1.3)	CTX-M-2	D	42
962	1 (1.3)	CTX-M-8	B1	0
1585	2 (2.6)	CTX-M-15	A	42
1642	1 (1.3)	CTX-M-15	B1	42
1727/446	41 (52.6)	CMY-2	B1	42
3994	14 (17.9)	CTX-M-8, CMY-2	A	42
5204	1 (1.3)	CTX-M-8	A	0
6618	4 (5.1)	CTX-M-2	D	42
NEW^b^	7 (8.9)	CTX-M-8	A, B2	0, 42


*STs are linked to the number of isolates belonging to each lineage as well as to the *bla* genes associated, the phylogenetic group and the day of feedlot each ST was identified. ^*a*^ST/CC, Sequence Type/Clonal Complex. ^*b*^New allelic combination: *adk*(295), *fumC*(54), *gyrB*(535), *icd*(767), *mdh*(260), *recA*(40), *purA*(83).*

## Discussion

Seventy-four (94.9%) *E. coli* isolates presented a multidrug-resistant antibiotype (MRAb) according to the antimicrobial susceptibility test, with the exception of the isolate 0.74E1, recovered on day 0 from animal #74, and the isolates 42.120E2, 42.127E1, 42.140E1, obtained on day 42 from animals #120, #127 and #140. Interestingly, all the non-MRAb *E. coli* were associated with *bla*_CTX-M-8_ gene (Figure 1). The high percentage of MRAb isolates illustrates the potential for spread of AR bacteria through a flock. Studies have already reported that the resistance rate to some antimicrobials rises during cattle or pig feedlot because of antimicrobial usage (Benedict et al., 2015; Gibbons et al., 2016; Weinroth et al., 2018). However, a Canadian study surveyed sheep flocks over a 1-year period and found no significant difference between the initial and the final visits (Scott et al., 2012), which is in disagreement with this study.

No isolate presented resistance to ertapenem (Table 1), which could be related to the fact that carbapenems are not approved for use in animals (OIE, 2018). The detection of AR *E. coli* in 55 animals after feedlot, in comparison to eight animals on day 0, indicates a selection pressure acting on the flock. Beta-lactams, florfenicol, macrolides, quinolones, tetracycline, and trimethoprim/sulfamethoxazole are administered in sheep (OIE, 2018). In fact, some animals included in this study received florfenicol or trimethoprim/sulfamethoxazole, and this could explain the presence of the AR *E. coli* because of direct or co-selection of resistance determinants in the GIT of the animals (Collignon et al., 2016; Makita et al., 2016; Knudsen et al., 2018).

The set of genes codifying beta-lactamase enzymes carried by the *E. coli* isolated on the 2 days of analysis was diverse. On day 0, 7.1% (8/112 animals) of the sampled lambs presented *E. coli* harboring some *bla*_CTX-M_-variant. However, after 42 days of feedlot the majority of *E. coli* isolated (53/78, 68.0% of the total) harbored the *bla*_CMY -2_ gene, comprising essentially the two great clusters of the dendrogram and the ST/CC 1727/446 and ST 3994 (Figure 1). Besides, *bla*_CMY -2_, the *bla*_CTX-M-2_ gene was detected only on day 42, while *bla*_CTX-M-14_ was detected in isolates recovered only on day 0 (Table 2). It seems that the first two genes entered into the flock during feedlot by some external factor such as surrounding animals, insects, or the environment (Blaak et al., 2015; Huijbers et al., 2015; Solà-Ginés et al., 2015), and the latter disappeared during feedlot perhaps because of competition between the *bla*_CTX-M-14_-carrying *E. coli* and other more successful strains, possibly the *bla*_CMY -2_-carrying *E. coli*. On the other hand, *bla*_CTX-M-8_ and *bla*_CTX-M-15_ were present on the first day of feedlot and persisted until the end (Table 2), which is clearly not linked to the maintenance of isolates into the feedlot, since the CTX-M-8- and the CTX-M-15-producing *E. coli* isolated on days 0 and 42 are not clonally related by PFGE or MLST (Figure 1). However, the majority of *bla*_CTX-M-8_ detected in isolates from day 42 are harbored by IncI1 plasmids of ∼97 kb, the same as two detected on day 0, which illustrates the maintenance and spreading of that plasmid through the feedlot. On the other hand, the *bla*_CTX-M-15_ gene identified in three *E. coli* recovered on day 42 probably entered the feedlot at some point since they are harbored by plasmid IncHI2 of ∼335 kb, differently from the *bla*_CTX-M-15_ harbored by an IncFII of ∼97 kb on day 0 (Table 3). Remarkably, some animals (2, 16, 19A, 19B, 20, 32, 50, 54, 57, 100, 111, 120, 140) carried more than one CMY-2 or CTX-M-producing *E. coli* on day 42, which are also present in other animals (Figure 1 and Table 2), which demonstrates the exchanging of commensal GIT bacteria among animals in the feedlot.

The use of a 3GC to enrich medium for recovery of *E. coli* from the feces of broilers induced a positivity of 99% of the samples containing *bla*_CMY -2_- and/or *bla*_CTX-M_-isolates (Verrette et al., 2019), which could be the explanation for the high percentage of such *E. coli* in our study. The *bla*_CMY -2_ gene has been reported as frequent in *E. coli* isolates causing urinary tract infections in Brazil (Rocha D. A. C. et al., 2016), and CMY-2- and CTX-M-producing *E. coli* were already isolated from poultry and buffalo in the country (Aizawa et al., 2014; Casella et al., 2018; Hoepers et al., 2018) but never in sheep. Apart from the prevalence of isolates presenting the *bla*_CMY -2_ gene, the occurrence of *bla*_CTX-M-14_- and *bla*_CTX-M-15_-carrying *E. coli* in this study is remarkable. Those genes are the dominant *bla*_CTX-M_ variants in most regions worldwide, concerning isolates from human infections and food-producing or companion animals (Zhao and Hu, 2013; Bevan et al., 2017; Chong et al., 2018; Dandachi et al., 2018). This means that the studied lambs represent a potential source of hard-to-treat infections caused by *E. coli* or at least a reservoir of important AR genes that could reach human pathogens. The *bla*_CTX-M-8_ gene was the second most detected in the studied population after *bla*_CMY -2_, present on both first and last days of feedlot (Table 2). CTX-M-8 was firstly identified in Brazil (Bonnet et al., 2000) and is still frequent in isolates from food-producing animals and meat in the country (Fernandes et al., 2016; Ferreira et al., 2016). However, it is thought to have a relatively low prevalence in other territories and is supposed to be transmitted by travelers or contaminated food (Dhanji et al., 2010; Egervärn et al., 2014; Eller et al., 2014).

Both genes *aac(3)-IIa* and *ant(2*″*)-Ia* codify resistance to gentamicin, and are present in plasmids (Ramirez and Tolmasky, 2010; Norris and Serpersu, 2013; Cox et al., 2015). In this study, *aac*(*3*)*-IIa* clearly predominated in relation to *ant(2*″*)-Ia* (Table 2). Notably, both genes reported here are clearly related to *E. coli* associated with infections (Miró et al., 2013; Fernández-Martínez et al., 2015). Resistance to phenicols was detected on the first and last days of feedlot, with *floR* and *cmlA* present in higher frequencies than the *cat* gene (Figure 1 and Table 2). A Portuguese study found only *cmlA* in *E. coli* isolated from sheep (Ramos et al., 2013), and a Brazilian study carried out with *Salmonella* Typhimurium isolated from humans and food revealed *floR* associated with food isolates and the *cat* gene associated with human *Salmonella* (Almeida et al., 2018). Furthermore, *cmlA* has already been reported in *E. coli* from chicken meat in the country (Casella et al., 2017a). Sixty-five isolates (83.3%) presented resistance to trimethoprim/sulfamethoxazole, but more than 60% of the *E. coli* presented at least one of the *sul* genes screened while 33.3% presented some *dfr* gene. Both *sul1* and *sul2* have been detected in *E. coli* isolated from sheep in Portugal (Ramos et al., 2013), and those genes have already been reported in *E. coli* isolated from clinical specimens (Oliveira-Pinto et al., 2017), chicken meat (Casella et al., 2015) and even surface water (Canal et al., 2016) in Brazil, but once again we know nothing about the subject in sheep. Resistance to tetracycline was detected during the entire feedlot stay of the lambs, with *tetA* and *tetB* detected on days 0 and 42, with considerable predominance of the first (Figure 1 and Table 2). Interestingly, the isolate 42.120E1 carried *tetA* and *tetB*, which is unexpected since both express the same tetracycline efflux mechanism (Thaker et al., 2010). *tetA* and *tetB* have already been detected in high frequencies in *E. coli* isolated from sheep (Ramos et al., 2013). The rising in the content of genes codifying resistance to tetracycline has been observed during bovine feedlot (Weinroth et al., 2018), but to our knowledge, there is no report of such an event concerning resistance to other antimicrobial classes in general, as observed in this study. In fact, the use of tetracyclines and trimethoprim/sulphonamides in sheep has already been reported as presenting a significant association with tetracycline resistance (Scott et al., 2012), and the *tetA* gene was positively associated with *bla*_CMY -2_ after ceftiofur followed chlortetracycline treatment in cattle (Kanwar et al., 2013), which is in agreement with our study. The *qnrB* gene was detected in six isolates recovered only on day 42, with all but one presenting intermediate resistance to the quinolones (Figure 1 and Table 2). A Chinese study reported *qnrB* as low-frequency among the genes detected in *E. coli* recovered from swine (Liu et al., 2018), and a recent study conducted in Brazil showed *E. coli* isolates carrying *qnrB* associated with the genes *bla*_CTX-M-2_ and *bla*_CMY -2_ in poultry (Ferreira et al., 2019). In our study, resistance to quinolones had little importance as a disseminated mechanism through the feedlot. Therefore, the presence of such genes codifying resistance to different antimicrobial classes in commensal isolates of food-producing animals as lambs raises public health concerns. The occurrence of MRAb *E. coli* in the studied lambs may be caused by the presence of animals and insects carrying these bacteria in the surroundings of the feedlot or even the environment (Blaak et al., 2015; Huijbers et al., 2015; Chong et al., 2018). Since we have collected feces from 112/140 flock animals, another possibility is a lamb not sampled as the source of that *E. coli*. Indeed, *bla*_CMY -2_-*floR*-*tetA*-*sul2*-harboring plasmids have already been identified in food-producing animals (Fernández-Alarcón et al., 2011) and could represent a similarity found in this study.

Regarding virulence genes, most isolates presented only *fimH* (Table 4), which is related to adhesion and is necessary for GIT colonization (Waksman and Hultgren, 2009). The absence of other virulence genes is not surprising, considering that the *E. coli* were isolated from feces of healthy animals and represent the GIT microbiota of the lambs. Instead of a known source of STEC strains in Brazil (Vettorato et al., 2009), sheep studied here did not present any evidence of carrying diarrheagenic *E. coli* (DEC). Nevertheless, all isolates were primarily selected from stools with the 3GC ceftiofur, which could represent a bias in the absence of STEC, EAEC or EPEC strains since such DEC could be present but do not carry genes for 3GC-resistance. The majority of the isolates (61.5%) belong to the phylogenetic group B1, 27.0% were classified as A, and 11.5% belong to phylogroups B2 or D (Figure 1 and Table 4). These results are in agreement with another study (Ramos et al., 2013), in which 61.1% of *E. coli* isolated from sheep were classified as phylogroup B1, 31.5% were phylogroup A, and 7.4% as phylogroups B2 or D. Traditionally, phylogenetic groups A and B1 are associated with commensal *E. coli*, while B2 and D with pathogenic isolates (Clermont et al., 2000), which is also in concordance with the few virulence genes detected.

Although the CMY-2-producers were distributed in different clusters according to *Xba*I-PFGE and belong to two different lineages according to MLST, the IncA/C plasmid of about 170 kb was confirmed as responsible for *bla*_CMY -2_ mobilization. This fact also illustrates the dissemination of that plasmid through the feedlot, which was indeed related to *bla*_CMY -2_ mobilization in food-producing animals and meat before, suggesting spread of the plasmid worldwide and in Brazil (Guo et al., 2014; Casella et al., 2017b; Dame-Korevaar et al., 2017). *bla*_CTX-M-8_ was carried by an IncI1 plasmid of ∼97 kb in isolates recovered on days 0 and 42, which seems to be responsible for the maintenance of that gene in the feedlot during the period analyzed. *bla*_CTX-M-8_-IncI1 plasmids have already been reported in *E. coli* isolated from humans, wastewater, food-producing animals and meat, and appear to be more responsible for the mobilization of that gene in several countries, including Brazil (Ferreira et al., 2014b; Dropa et al., 2016; Norizuki et al., 2017; Casella et al., 2018; Dantas Palmeira et al., 2018). The *bla*_CTX-M-15_ gene was carried by very different plasmids on the first and last days of feedlot (Table 3), which means that the *bla*_CTX-M-15_-IncFII present on day 0 probably disappeared and the *bla*_CTX-M-15_-IncHI2 entered the feedlot at any time point during the period. Since both plasmids are carried by extremely different *E. coli*, according to *Xba*I-PFGE and MLST methodologies (Figure 1), it seems that the change on plasmids responsible for *bla*_CTX-M-15_ mobilization was due to the disappearance and entry of respective strains into the feedlot, contrary to what happened to the *bla*_CTX-M-8_-IncI1 plasmids of about 97 kb mentioned above. IncHI2 plasmids have also been reported as responsible for mobilization of *bla*_CTX-M-15_ in several *Enterobacteriaceae* species isolated from humans or animals (Kariuki et al., 2015; Haenni et al., 2016) and have been detected in 3/4 of the CTX-M-15-producing *E. coli* in this study. The CTX-M-2-producers identified in this study seem to carry the *bla*_CTX-M-2_ inserted into the chromosome. This is not a rare event nowadays and is plausible since it has already been reported in *E. coli* isolated from chickens and chicken meat in Brazil (Ferreira et al., 2014a; Casella et al., 2018). In addition to that, *bla*_CTX-M-2_-carrying *E. coli* were isolated just on day 42 and were clonally related by *Xba*I-PFGE and MLST, with the exception of isolate 42.2E2 (Figure 1). Finally, we could not detect the plasmid linked to *bla*_CTX-M-14_, and this gene has already been described inserted into the chromosome (Hamamoto et al., 2016; Hamamoto and Hirai, 2019), which could be the explanation for the present isolates. Further studies are required to elucidate this subject.

*Xba*I-PFGE typing grouped most of the *bla*_CMY -2_-carrying *E. coli* in the two major clusters, composed of 37 and 13 *E. coli* that carry essentially *bla*_CMY -2_-*aac(3)-IIa*-*tetA*-*sul1*-*sul2*-*floR*-*cmlA*, with exceptions, belonging to phylogroups B1-ST/CC 1727/446 or A-ST 3994, respectively (Figure 1). Strains belonging to the later cluster additionally carry the *dfr Ia* and *dfr XII* genes. This finding indicates that two strains have spread among animals throughout the feedlot, but all harboring the same *bla*_CMY -2_-carrying plasmid as mentioned above. Interestingly, two lineages were detected carrying different *bla* genes, such as ST/CC 58/155 presenting *bla*_CTX-M-8_ or *bla*_CTX-M-14_ and ST 3994 presenting *bla*_CTX-M-8_ or *bla*_CMY -2_, and both groups have a considerable relationship within isolates (Figure 1). *E. coli* ST/CC 58/155 has already been reported harboring *bla*_CTX-M-14_ and others from clinical specimens and healthy people in several countries (Gerhold et al., 2016; Kawamura et al., 2017). In Brazil, this lineage has already been reported carrying *bla*_CTX-M-8_ or *bla*_CMY -2_ in dogs, and the *bla*_CTX-M-8_ gene was also harbored by an IncI1 plasmid (Melo et al., 2018), as in this study. Furthermore, the same Brazilian study showed an *E. coli* phylogroup D-ST 457 isolated from a diseased dog carrying the *bla*_CTX-M-2_ inserted in the chromosome, the same as the only CTX-M-2-producing isolate ST 457 in this study, which demonstrates the presence of that clone in different animals in the country. Contrary to the clonality described above regarding ST/CC 58/155, the *E. coli* ST/CC 1727/446 isolated in this study carry only *bla*_CMY -2_, but isolates were not clonally related according to *Xba*I-PFGE typing (Figure 1). This could represent micro-evolution occurring in the *E. coli* strains in the feedlot during the period of 42 days. The new combination of alleles (new ST) found in seven related *bla*_CTX-M-8_-carrying isolates was the unique lineage recovered on days 0 and 42, which means that the clone remained in the studied feedlot lambs carrying the same *bla*_CTX-M-8_-IncI1 plasmid (Figure 1 and Table 5).

## Conclusion

In conclusion, feedlot lambs act as reservoirs of commensal multidrug-resistant *E. coli*, and those AR genes or bacteria can reach humans through the food chain. The presence of *bla*_CTX-M-14_ and *bla*_CTX-M-15_ deserves special attention since they are the genes most related to human infections worldwide. To the best of our knowledge, this is the first report of *bla*_CTX-M-14_ in *Enterobacteriaceae* isolated from food-producing animals in Brazil. Additionally, *E. coli* ST lineages and plasmids harboring the *bla* genes detected have already been identified in humans, animals, meat and the environment, which demonstrates the concern for their dissemination and for public health. Further studies are needed in order to determine the reasons for the success of the *bla*_CMY -2_-*aac(3)*-*IIa*-*tetA*-*sul1*-*sul2*-*floR*-*cmlA*-carrying *E. coli* in the studied feedlot. To the best of our knowledge, this is the first study reporting such a broad characterization of antimicrobial resistant *E. coli* isolated from sheep.

## Data Availability

The datasets generated for this study can be found in GenBank, MK896925 to MK896944, and MK917695 to MK917713.

## Ethics Statement

Ethics Committee approval number FOA00845-2017. Universidade Estadual Paulista (UNESP) “Júlio de Mesquita Filho,” campus de Araçatuba, Faculdade de Medicina Veterinária.

## Author Contributions

TC, MN, and LM designed the study. KG, JF, LDA, and CS performed all the laboratorial experiments. RB handled with the animals and their stools. TC, MN, LM, JP, and MM wrote and revised the whole manuscript.

## Conflict of Interest Statement

The authors declare that the research was conducted in the absence of any commercial or financial relationships that could be construed as a potential conflict of interest.

## Footnotes

1 http://blast.ncbi.nlm.nih.gov/

2 https://pubmlst.org/bigsdb?db=pubmlst_mlst_seqdef
