# Supplementary material for: Dissemination of Multidrug-Resistant Commensal Escherichia coli in Feedlot Lambs in Southeastern Brazil
Source: Front Microbiol. 2019 Jun 25;10:1394. doi: 10.3389/fmicb.2019.01394 (PMC6603138; doi:10.3389/fmicb.2019.01394)
Supplement: Supplementary file 1 [file Table_1.docx]

**Supplemental Table 1.** Set of primers used in this study.

| **Gene target** | **Primer sequence (5' - 3')** | **Reference** |
| --- | --- | --- |
| *bla*_CMY-2_ | ATGATGAAAAAATCGTTATGC | (Mammeri et al., 2010) |
|  | TTATTGCAGCTTTTCAAGAATGC |  |
| group *bla*_CTX-M-1_ | TTAGGAARTGTGCCGCTGYA | (Dallenne et al., 2010) |
|  | CGATATCGTTGGTGGTRCCAT |  |
| group *bla*_CTX-M-2_ | CGTTAACGGCACGATGAC | (Dallenne et al., 2010) |
|  | CGATATCGTTGGTGGTRCCAT |  |
| group *bla*_CTX-M-9_ | TCAAGCCTGCCGATCTGGT | (Dallenne et al., 2010) |
|  | TGATTCTCGCCGCTGAAG |  |
| group *bla*_CTX-M-8/-25_ | AACRCRCAGACGCTCTAC | (Dallenne et al., 2010) |
|  | TCGAGCCGGAASGTGTYAT |  |
| *aac(3)-Ia* | GACATAAGCCTGTTCGGTT | (Akers et al., 2010) |
|  | CCCGCTTTCTCGTAGCA |  |
| *aac(3)-IIa* | ATGCATACGCGGAAGGC | (Akers et al., 2010) |
|  | TGCTGGCACGATCGGAG |  |
| *aac(6′)-Ib* | TATGAGTGGCTAAATCGAT | (Akers et al., 2010) |
|  | CCCGCTTTCTCGTAGCA |  |
| *aac(6')-Ih* | TGCCGATATCTGAATC | (Akers et al., 2010) |
|  | ACACCACACGTTCAG |  |
| *aph(3’)-Ia* | CGAGCATCAAATGAAACTGC | (Akers et al., 2010) |
|  | GCGTTGCCAATGATGTTACAG |  |
| *aph(3')-VI* | CGGAAACAGCGTTTTAGA | (Akers et al., 2010) |
|  | TTCCTTTTGTCAGGTC |  |
| *ant(2'')-Ia* | ATCTGCCGCTCTGGAT | (Akers et al., 2010) |
|  | CGAGCCTGTAGGACT |  |
| *qrnA* | AGAGGATTTCTCACGCCAGG | (Cattoir et al., 2007) |
|  | TGCCAGGCACAGATCTTGAC |  |
| *qrnB* | GGMATHGAAATTCGCCACTG | (Cattoir et al., 2007) |
|  | TTTGCYGYYCGCCAGTCGAA |  |
| *qrnS* | GCAAGTTCATTGAACAGGGT | (Cattoir et al., 2007) |
|  | TCTAAACCGTCGAGTTCGGCG |  |
| *qrnC* | GGGTTGTACATTTATTGAATC | (Wang et al., 2009) |
|  | TCCACTTTACGAGGTTCT |  |
| *qrnD* | TTTTCGCTAACTAACTCGC | (Zhao et al., 2010) |
|  | GAAAGGATAAACAGGCAAAT |  |
| *qepA* | CGGCGGCGTGTTGCTGGAGTTCTT | (Ma et al., 2009) |
|  | CCGACAGGCCCACGACGAGGATGC |  |
| *oqxAB* | CCCTGGACCGCACATAAAG | (Zhao et al., 2010) |
|  | AAAGAACAAGATTCACCGCAAC |  |
| *tet(A)* | GCTACATCCTGCTTGCCTTC | (Ng et al., 2001) |
|  | CATAGATCGCCGTGAAGAGG |  |
| *tet(B)* | TTGGTTAGGGGCAAGTTTTG | (Ng et al., 2001) |
|  | GTAATGGGCCAATAACACCG |  |
| *tet(C)* | CTTGAGAGCCTTCAACCCAG | (Ng et al., 2001) |
|  | ATGGTCGTCATCTACCTGCC |  |
| *dfrIa* | GTGAAACTATCACTAATGG | (Navia et al., 2003) |
|  | TTAACCCTTTTGCCAGATTT |  |
| *dfrVII* | TTGAAAATTTCATTGATTG | (Navia et al., 2003) |
|  | TTAGCCTTTTTTCCAAATCT |  |
| *dfrXII* | GGTGSGCAGAAGATTTTTCGC | (Navia et al., 2003) |
|  | TGGGAAGAAGGCGTCACCCTC |  |
| *Sul1* | CGGCGTGGGCTACCTGAACG | (Vrints et al., 2009) |
|  | GCCGATCGCGTGAAGTTCCG |  |
| *Sul2* | GCGCTCAAGGCAGATGGCATT | (Vrints et al., 2009) |
|  | GCGTTTGATACCGGCACCCGT |  |
| *floR* | CACGTTGAGCCTCTATATGG | (Ng et al., 1999) |
|  | ATGCAGAAGTAGAACGCGAC |  |
| *cat* | CCACCGTTGATATATCCC | (Guerra et al., 2001) |
|  | CCTGCCACTCATCGCAGT |  |
| *cmlA* | CCGCCACGGTGTTGTTGTTATC | (Van et al., 2008) |
|  | CACCTTGCCTGCCCATCATTAG |  |
| *fimH* | TGCAGAACGGATAAGCCGTGG | (Johnson and Stell, 2000) |
|  | GCAGTCACCTGCCCTCCGGT |  |
| *papEF* | GCAACAGCAACGCTGGTTGCATCAT | (Yamamoto et al., 1995) |
|  | AGAGAGAGCCACTCTTATACGGACA |  |
| *papG I* | TTAGCTGGATGGCACAATG | (Mora et al., 2013) |
|  | TTGTCCATGTATCCCATTCAT |  |
| *papG II* | GGGCATTGCTACGGTAACCTG | (Mora et al., 2013) |
|  | CGCTATTAATAGACAGATCACC |  |
| *papG III* | CGGCAACTTTAAGCTATGTG | (Mora et al., 2013) |
|  | TGTACCATCTCATCGTTGTCTC |  |
| *sfa/focDE* | CTCCGGAGAACTGGGTGCATCTTAC | (Le Bouguénec et al., 1992) |
|  | CGGAGGAGTAATTACAAACCTGGCA |  |
| *sfaS* | GTGGATACGACGATTACTGTG | (Johnson and Stell, 2000) |
|  | CCGCCAGCATTCCCTGTATTC |  |
| *focG* | CAGCACAGGCAGTGGATACGA | (Johnson and Stell, 2000) |
|  | GAATGTCGCCTGCCCATTGCT |  |
| *afa/draBC* | GCTGGGCAGCAAACTGATAACTCTC | (Le Bouguénec et al., 1992) |
|  | CATCAAGCTGTTTGTTCGTCCGCCG |  |
| *nfaE* | GCTTACTGATTCTGGGATGGA | (Johnson and Stell, 2000) |
|  | CGGTGGCCGAGTCATATGCCA |  |
| *kpsMT II K1* | TAGCAAACGTTCTATTGGTGC | (Johnson and Stell, 2000) |
|  | CATCCAGACGATAAGCATGAGCA |  |
| *kpsMT II K5* | CAGTATCAGCAATCGTTCTGTA | (Johnson and Stell, 2000) |
|  | CATCCAGACGATAAGCATGAGCA |  |
| *hlyA* | AACAAGGATAAGCACTGTTCTGGCT | (Yamamoto et al., 1995) |
|  | ACCATATAAGCGGTCATTCCCGTCA |  |
| *cnf1* | AAGATGGAGTTTCCTATGCAGGAG | (Yamamoto et al., 1995) |
|  | CATTCAGAGTCCTGCCCTCATTATT |  |
| *cdtB* | GAAAGTAAATGGAATATAAATGTCCG | (Tóth et al., 2003) |
|  | AAATCACCAAGAATCATCCAGTTA |  |
| *sat* | GCAGCTACCGCAATAGGAGGT | (Johnson et al., 2003) |
|  | CATTCAGAGTACCGGGGCCTA |  |
| *vat* | GAACACAGTTCATCTGATCTCC | (Parham et al., 2005) |
|  | GAATATATCAAATTGGTCCCCC |  |
| *fyuA* | TGATTAACCCCGCGACGGGAA | (Johnson et al., 2003) |
|  | CGCAGTAGGCACGATGTTGTA | (Russo et al., 1999) |
| *iutA* | GGCTGGACATCATGGGAACTGG | (Johnson et al., 1998) |
|  | CGTCGGGAACGGGTAGAATCG |  |
| *iroN* | AAGTCAAAGCAGGGGTTGCCCG | (Johnson et al., 2000) |
|  | GACGCCGACATTAAGACGCAG |  |
| *gadA* | GATGAAATGGCGTTGGCGCAAG | (Doumith et al., 2012) |
|  | GGCGGAAGTCCCAGACGATATCC |  |
| *chuA* | ATGATCATCGCGGCGTGCTG | (Doumith et al., 2012) |
|  | AAACGCGCTCGCGCCTAAT |  |
| *yjaA* | TGTTCGCGATCTTGAAAGCAAACGT | (Doumith et al., 2012) |
|  | ACCTGTGACAAACCGCCCTCA |  |
| TSPE4.C2 | GCGGGTGAGACAGAAACGCG | (Doumith et al., 2012) |
|  | TTGTCGTGAGTTGCGAACCCG |  |

Akers, K. S., Chaney, C., Barsoumian, A., Beckius, M., Zera, W., Yu, X., et al. (2010). Aminoglycoside resistance and susceptibility testing errors in Acinetobacter baumannii-calcoaceticus complex. *J. Clin. Microbiol.* 48, 1132–8. doi:10.1128/JCM.02006-09.

Cattoir, V., Poirel, L., Rotimi, V., Soussy, C. J., and Nordmann, P. (2007). Multiplex PCR for detection of plasmid-mediated quinolone resistance qnr genes in ESBL-producing enterobacterial isolates. *J Antimicrob Chemother* 60, 394–397. doi:10.1093/jac/dkm204.

Dallenne, C., da Costa, A., Decré, D., Favier, C., and Arlet, G. (2010). Development of a set of multiplex PCR assays for the detection of genes encoding important β-lactamases in Enterobacteriaceae. *J. Antimicrob. Chemother.* 65, 490–495. doi:10.1093/jac/dkp498.

Doumith, M., Day, M. J., Hope, R., Wain, J., and Woodford, N. (2012). Improved multiplex PCR strategy for rapid assignment of the four major Escherichia coli phylogenetic groups. *J Clin Microbiol* 50, 3108–3110. doi:10.1128/JCM.01468-12.

Guerra, B., Soto, S. M., Argüelles, J. M., and Mendoza, M. C. (2001). Multidrug resistance is mediated by large plasmids carrying a class 1 integron in the emergent Salmonella enterica serotype [4,5,12:i:-]. *Antimicrob. Agents Chemother.* 45, 1305–1308. doi:10.1128/AAC.45.4.1305-1308.2001.

Johnson, J. R., Brown, J. J., Carlino, U. B., and Russo, T. A. (1998). Colonization with and acquisition of uropathogenic Escherichia coli as revealed by polymerase chain reaction-based detection. *J. Infect. Dis.* 177, 1120–4. Available at: http://www.ncbi.nlm.nih.gov/pubmed/9534997.

Johnson, J. R., Gajewski, A., Lesse, A. J., and Russo, T. A. (2003). Extraintestinal Pathogenic Escherichia coli as a Cause of Invasive Nonurinary Infections. *J. Clin. Microbiol.* 41, 5798–5802. doi:10.1128/JCM.41.12.5798-5802.2003.

Johnson, J. R., Russo, T. A., Tarr, P. I., Bilge, S. S., Jr, J. C. V., Stell, A. L., et al. (2000). Molecular Epidemiological and Phylogenetic Associations of Two Novel Putative Virulence Genes , iha and iroN E . coli , among Escherichia coli Isolates from Patients with Urosepsis Molecular Epidemiological and Phylogenetic Associations of Two Novel Putat. *Infect. Immun.* 68, 3040–3047. doi:10.1128/IAI.68.5.3040-3047.2000.Updated.

Johnson, J. R., and Stell, A. L. (2000). Extended virulence genotypes of Escherichia coli strains from patients with urosepsis in relation to phylogeny and host compromise. *J. Infect. Dis.* 181, 261–272. doi:10.1086/315217.

Le Bouguénec, C., Archambaud, M., and Labigne, A. (1992). Rapid and Specific Detection of the pap, afa, and sfa Adhesin-Encoding Operons in Uropathogenic Escherichia coli Strains by Polymerase Chain Reaction. *J. Clin. Microbiol.* 30, 1189–1193. doi:10.1093/jac/dkl326.

Ma, J., Zeng, Z., Chen, Z., Xu, X., Wang, X., Deng, Y., et al. (2009). High prevalence of plasmid-mediated quinolone resistance determinants qnr, aac(6’)-Ib-cr, and qepA among ceftiofur-resistant Enterobacteriaceae isolates from companion and food-producing animals. *Antimicrob Agents Chemother* 53, 519–524. doi:10.1128/AAC.00886-08.

Mammeri, H., Guillon, H., Eb, F., and Nordmann, P. (2010). Phenotypic and biochemical comparison of the carbapenem-hydrolyzing activities of five plasmid-borne AmpC β-lactamases. *Antimicrob. Agents Chemother.* 54, 4556–4560. doi:10.1128/AAC.01762-09.

Mora, A., Viso, S., Lopez, C., Alonso, M. P., Garcia-Garrote, F., Dabhi, G., et al. (2013). Poultry as reservoir for extraintestinal pathogenic Escherichia coli O45:K1:H7-B2-ST95 in humans. *Vet Microbiol* 167, 506–512. doi:10.1016/j.vetmic.2013.08.007.

Navia, M. M., Ruiz, J., Sanchez-Cespedes, J., and Vila, J. (2003). Detection of dihydrofolate reductase genes by PCR and RFLP. *Diagn. Microbiol. Infect. Dis.* 46, 295–298. doi:10.1016/S0732-8893(03)00062-2.

Ng, L.-K., Martin, I., Alfa, M., and Mulvey, M. R. (2001). Multiplex PCR for the detection of tetracycline resistant genes. *Mol Cell Probes* 15, 209–215. doi:10.1006/mcpr.2001.0363.

Ng, L.-K., Mulvey, M. R., Martin, I., Peters, G. A., and Johnson, W. (1999). Genetic characterization of antimicrobial resistance in Canadian isolates of Salmonella serovar Typhimurium DT104. *Antimicrob. Agents Chemother.* 43, 3018–3021. doi:10.1007/978-3-642-16499-6_10.

Parham, N. J., Pollard, S. J., Desvaux, M., Scott-Tucker, A., Liu, C., Fivian, A., et al. (2005). Distribution of the serine protease autotransporters of the Enterobacteriaceae among extraintestinal clinical isolates of Escherichia coli. *J Clin Microbiol* 43, 4076–4082. doi:10.1128/JCM.43.8.4076-4082.2005.

Russo, T. A., Carlino, U. B., Mong, A., and Jodush, S. T. (1999). Identification of Genes in an Extraintestinal Isolate of Escherichia coli with Increased Expression after Exposure to Human Urine Identification of Genes in an Extraintestinal Isolate of Escherichia coli with Increased Expression after Exposure to Human U. *Infect. Immun.* 67, 5306–5314.

Tóth, I., Hérault, F., Beutin, L., and Oswald, E. (2003). Production of cytolethal distending toxins by pathogenic Escherichia coli strains isolated from human and animal sources: Establishment of the existence of a new cdt variant (type IV). *J. Clin. Microbiol.* 41, 4285–4291. doi:10.1128/JCM.41.9.4285-4291.2003.

Van, T. T. H., Chin, J., Chapman, T., Tran, L. T., and Coloe, P. J. (2008). Safety of raw meat and shellfish in Vietnam: An analysis of Escherichia coli isolations for antibiotic resistance and virulence genes. *Int. J. Food Microbiol.* 124, 217–223. doi:10.1016/j.ijfoodmicro.2008.03.029.

Vrints, M., Mairiaux, E., Van Meervenne, E., Collard, J. M., and Bertrand, S. (2009). Surveillance of antibiotic susceptibility patterns among Shigella sonnei strains isolated in Belgium during the 18-year period 1990 to 2007. *J. Clin. Microbiol.* 47, 1379–1385. doi:10.1128/JCM.02460-08.

Wang, M., Guo, Q., Xu, X., Wang, X., Ye, X., Wu, S., et al. (2009). New plasmid-mediated quinolone resistance gene, qnrC, found in a clinical isolate of Proteus mirabilis. *Antimicrob Agents Chemother* 53, 1892–1897. doi:10.1128/AAC.01400-08.

Yamamoto, S., Terai, A., Yuri, K., Kurazono, H., Takeda, Y., and Yoshida, O. (1995). Detection of urovirulence factors in Escherichia coli by multiplex polymerase chain reaction. *FEMS Immunol. Med. Microbiol.* 12, 85–90. Available at: http://www.ncbi.nlm.nih.gov/pubmed/8589667.

Zhao, J., Chen, Z., Chen, S., Deng, Y., Liu, Y., Tian, W., et al. (2010). Prevalence and dissemination of oqxAB in Escherichia coli isolates from animals, farmworkers, and the environment. *Antimicrob Agents Chemother* 54, 4219–4224. doi:10.1128/AAC.00139-10.
